# Size, skills, and suffrage: Motivated distortions in perceived formidability of political leaders

**DOI:** 10.1371/journal.pone.0188485

**Published:** 2017-12-21

**Authors:** Jill E. P. Knapen, Nancy M. Blaker, Thomas V. Pollet

**Affiliations:** 1 Section Social and Organizational Psychology, Department of Experimental and Applied Psychology, Vrije Universiteit Amsterdam, Amsterdam, The Netherlands; 2 Department of Work and Organizational Psychology, University of Amsterdam, Amsterdam, The Netherlands; 3 Department of Management, University of Otago, Dunedin, New Zealand; 4 Department of Psychology, Northumbria University, Newcastle upon Tyne, United Kingdom; University of Lethbridge, CANADA

## Abstract

Research shows that perception of physical size and status are positively associated. The current study was developed to replicate and extend earlier research on height perceptions of political leaders, indicating that supporters perceive their leaders as taller than non-supporters do, and winners are perceived as taller after the elections, while losers are perceived as shorter after the elections (winner/loser effects). Individuals use greater height and strength as indications of greater physical formidability. We hypothesized that in-group leaders’ height and strength, but not weight, would be overestimated more compared to out-group leaders’, and that this status-size association is not only driven by dominance, but also by prestige. We also tested whether previously found gender effects in estimates were due to using one’s own height as an anchor, and we used an improved methodological approach by relying on multiple measurements of physical formidability and a within-subject design for testing winner/loser effects. The results of a two-part longitudinal study (self-selected sample via voting advice website; *N*_*Wave1*_ = 2,011; *N*_*Wave2*_ = 322) suggest that estimated physical formidability of political leaders is affected by motivated perception, as prestige was positively associated with estimated formidability, and in-group leaders were estimated more formidable than out-group leaders. We conclude that distortions in judged formidability related to social status are the result of motivated social perception in order to promote group functioning and leadership. Although we did not replicate a winner-effect (greater estimations of formidability after winning the elections), we did find some evidence for a loser-effect. Earlier suggestions that men make larger estimations than women because of their own larger body size are not supported. Implications for theory and future research are discussed.

## Introduction

In traditional societies in Melanesia and Polynesia, high status individuals are referred to as ‘Big Men’ [1,2]. These Big Men are literally of tall stature, and their status is partially derived from them being good and brave warriors in intergroup conflicts and providing their followers with protection [2–4]. Previous research suggests that this association between physical formidability and social status holds a kernel of truth, especially for men. Ethnographic studies show that in many cultures, a relatively tall stature is associated with positions of power (e.g., [5–8]). In addition, research has shown positive effects of stature on fighting ability [9,10]. This association between height and social status is still relevant in modern Western society, as height is positively associated with career success, political success, and authority [11–13]. Perhaps unsurprisingly, these size-status associations are also present in our social perceptions: men of tall stature are perceived as being more competent [11,14], more leader-like [15], and are more attractive [16–18] (but see [19]) than men of short stature. Height and strength are components of one’s formidability, or how large and strong an individual is conceptualized to be [20]. However, Sell and colleagues [21] found that fighting ability was better predicted by upper-body strength than it was by body height. In this study, we hypothesize that the association between perceived formidability and perceived status is a strong, bidirectional one: Earlier findings show an effect of height on status, and we also expect an effect of status on height. Moreover, we argue that this association is not only driven by dominance, but also by prestige. Furthermore, we argue that distortions in judged formidability related to prestige are the result of motivated social perception, which could function to promote group functioning and leadership.

### Distortions in judged formidability

Do individuals make perceptual errors when it comes to judging physical formidability? When processing social information, the metaphors we use to construe our perceptions of others can be influenced by the way in which people are presented to us [22]. For example, embodiment research suggests that power is mentally represented in terms of size [23]: using a larger font or a vertically high position leads to greater perceptions of power of presented stimuli [23–26].

Indeed, social perception research shows that people associate status with physical size, especially in men [11,15]. Children [27,28], and even infants as young as 10 months old [29], already use size to mentally represent social dominance. Likewise, adults generally tend to see taller individuals as more dominant, intelligent, competent, persuasive, and as better leadership material overall than they do shorter individuals [15,16,30,31]. Furthermore, tall leaders are perceived as more charismatic than shorter leaders are [32], and taller US presidents are rated as having more ‘presidential greatness’ than their shorter counterparts [12]. Lastly, people tend to estimate a man’s height as greater when they are told he has a higher status as opposed to a lower status [33,34] or when he acts more dominantly instead of submissively [35].

### Motivated social perception of leaders

Psychological research has shown that our interpersonal perception is subject to all kinds of biases and errors [36]. Haselton and Funder [37] argued that these perceptual errors may have been shaped via both natural and sexual selection to serve purposes which would have been relevant to us throughout human evolution, producing “a mind that produces judgments that are sufficiently accurate given cognitive and informational constraints, not perfect” (pp. 31). In the interpersonal domain, using a heuristic for a ‘quick and dirty’ judgment of others would suffice for the purpose of the anticipated interaction with another individual. Even though the use of a heuristic might lead to erroneous perceptions, error management theory posits that such heuristics might still occur because certain errors are less costly than other errors made when responding with inappropriate behavior when not using the heuristic [38,39].

The status-related distortions in judgments of formidability described above can be explained from both a heuristics view and a motivated perception point of view. As the association between size and status is at least partly grounded in reality, it is presumably a good heuristic for making ‘quick and dirty’ judgments about others: research has shown that height is positively associated with actual income [40], career success [11,41], and occupation of high status positions [42,43]. Therefore, judging high-status people as more formidable (or the other way around) would probably result in what Haselton and Funder [37] defined as ‘useful degree of accuracy’. The association is strong enough that the estimation is probably right and moreover, the costs of overestimating formidability would probably be less than the costs of underestimating it. This could make the perception sufficient for short-term survival and mating purposes, even when it is not entirely accurate.

Another potential cause for perceptual errors is motivated perception: individuals tend to see others in a way that confirms their expectations [44–46]. Perceptual errors have been shown in perception research for zero-acquaintance judgments as well as judgments of people with whom we (expect to) have longer interactions or even relationships (e.g., [46–49]). Indeed, social goals can distort our perceptions of both ourselves and others, which might facilitate our navigation through the social world [49–51]. We hypothesize that distortions in judgments of formidability of political leaders could have a motivational component with regards to defensive threat regulation and effective leadership. Lopez, McDermott, and Petersen [52] argued that humans have an ‘evolved coalitional psychology’ that enables and generates behavior in social and political matters like coalition formation and maintenance, trade, and war. One of the functions of this coalitional psychology is to deal with internal coalition dynamics like leadership and cooperation, as individuals within a coalition need to interact constructively to achieve common goals and share resources. This coalitional psychology is proposed to have strong implications for leadership, and leaders’ (perceived) representation of themselves and their group towards both enemies and allies.

Holbrook and Fessler [53] propose that “humans may have evolved efficient ways of representing group formidability in order to facilitate assessments of whether to fight, flee, or appease enemies” (pp 46). As leaders are (one of the most) visible members of the group, they arguably also play an important role in representing the formidability of the group. Having a formidable leader would be beneficial when dealing with other tribes, to emphasize the strength of the group as a desired ally and/or enemy to be avoided [54]. Moreover, Lopez et al. [52] suggest that as the leaders of our evolutionary past were most likely the initiators and representatives in dealing with other tribes, numerous changes in leadership position could send the message of an unstable group, which would be perceived as less desirable allies, both in politics and in trade. In terms of consistent leadership, it would benefit the group when this leader would not be challenged (often), and the other members of the group would endorse his position. Overestimation of the in-group leader’s formidability by the other group members could thus potentially allow for effective and stable leadership within a coalition.

### Overview of the current research

#### Overestimation of in-group leaders

If perceptual distortions of physical formidability are the result of a motivated social perception that goes above and beyond a mere heuristic, the association between status and size should be stronger when there are potential benefits to the perceiver. Therefore, we expect that in-group leaders will be perceived as taller and stronger (but not heavier) compared to out-group leaders, as a result of motivated perception underpinning effective coalitional psychology. In line with this argument, Sorokowski [55] showed that in most cases, presidential candidates in Poland were estimated to be taller by their supporters than by their opponents. Research has shown that the presence of in-group members can have a similar effect on the motivated perception of out-group threats: when individuals are surrounded by their in-group members (compared to alone), they perceive a threatening person from an out-group as relatively more distant [56] and relatively less formidable [57].

#### Prestige effects

Social perception research on height and status tends to focus on dominance-based status, while prestige-based status is understudied. Research by Henrich and colleagues has argued that there are by and large two behavioral pathways via which formidability can translate into social status; either through dominance or prestige [58,59]. Men can attain dominance by (aggressive) competition with other men, and men with greater physical strength have a greater likelihood to defeat their opponent [10, 60]. A second pathway via which men can achieve status is through accumulating prestige benefits: “the sharing of expertise or know-how to gain respect” [56] (pp 103). Perceived physical formidability is used as a cue for interpreting leadership skills [15,61], as men with greater physical formidability are better able to effectively regulate within-group processes [61,62] and to represent the group during collaborations or conflicts with other groups [2–4,15,27,54]. In this case, formidable men gain social status because they can provide benefits for the group (prestige), and not because they are able to physically enforce their will on others (dominance). If the status-size association is not only driven by dominance, but also by prestige, then being perceived as having expertise in your high-status function should have an effect on your perceived formidability (i.e. a motivated perception to see competent leaders as more formidable). For this study, we use perceived political skills as a measure for the perceived expertise (thus, prestige) of political leaders. We expect that when leaders are perceived as having good political skills, they will also be seen as taller and stronger (but not heavier).

#### Winner/loser effects

We also expect that perceptions of physical formidability in winners will be overestimated, while physical formidability in losers will be underestimated. For example, Holbrook and Fessler [53] showed that after Osama Bin Laden was killed, Americans estimated an Islamic terrorist to be smaller and weaker, while primes of victorious terrorist leaders caused the Islamic terrorists to be estimated larger and stronger. Likewise, Higham and Carment [63] found that, compared to height judgments before the elections, politicians who lost an election were judged as shorter, while the politician who won was judged as being taller. Higham and Carment attributed this change to the winner’s change in social status. However, research by Sorokowski [55] on the 2005 presidential elections in Poland suggests that these changes in height perceptions are due to the individual perceiver’s support as well as social status through reported electoral support rankings in the media. We expect that winners will be seen as taller and stronger (but not heavier) after the elections compared to before the elections, while we expect that losers will be seen as shorter and weaker (but not less heavy) after the elections compared to before the elections.

#### Effects of perceiver sex

Higham and Carment [63] showed that men tended to judge politicians as taller than women did. This finding was explained by men generally being taller than women are, and perceivers using their own height as an anchor for making estimations about the height of others. However, Higham and Carment [63] did not include actual height measurements of their participants to investigate this explanation. Another reason for this effect could be that men are more sensitive to formidability cues of other men, as competitive and aggressive activities are generally mostly carried out by males [64–67]. We expect that men will indeed make larger overestimations than women will make, and we will control for anchoring effects based on their own height and weight by asking participants to state their own height and weight (self-report), and by having multiple height- and weight perception measurements. By also including relative measurements as opposed to only absolute measurements, we can test the anchoring effect more critically, as absolute measures should be more sensitive to anchoring effects than relative measures.

#### Hypotheses

We examined our hypotheses in the political domain by asking participants to make estimations of political leaders during times of election. The coalitional group is represented by the political party with which one identifies. Political leaders are among the best examples of our current group leaders and as such, are often subject of leadership research concerning height. For example, Stulp and colleagues [12] found that although taller presidential candidates for the US elections are not more likely to actually win an election, taller candidates were more likely to get a larger share of the popular vote and were also more likely to be re-elected than their shorter opponents. As Sorokowski [53] noted, except for his own research on height perception of politicians in a European country (Poland) with a multi-party system (as opposed to the North-American two-party system), most of the height perception research on politicians has been done in North America. Studying the Dutch multi-party system thus adds extra knowledge to the current body of research, as we know comparatively little about perceptions of height in multiparty systems. Via a longitudinal two-part study, we tested the following predictions:

P1: In-group leaders will be estimated as taller compared to out-group leaders;
P2: In-group leaders will be estimated as stronger compared to out-group leaders;
P3: There will be no difference in weight estimations between in-group and out-group leaders;
P4: Perceived prestige of leaders will have a positive association with perceived height of leaders;
P5: Perceived prestige of leaders will have a positive association with perceived strength of leaders;
P6: Perceived prestige of leaders will not be significantly associated with perceived weight of leaders;
P7: Winners will be estimated as taller compared to losers;
P8: Winners will be estimated as stronger compared to losers;
P9: There will be no difference in weight estimations between winners and losers;
P10: Men will generally make larger estimations of height than women will make.
P11: Men will generally make larger estimations of strength than women will make.

We will conduct exploratory analyses to examine whether men make larger estimations of weight than women make. Both the anchoring theory and sensitivity to opponents’ physical formidability theory predict larger height and strength estimations by men, while only the anchoring theory predicts larger weight estimations by men.

In conclusion, we examine whether distortions in judged formidability related to social status are the result of motivated social perception in order to promote group functioning and leadership. We will focus in particular on falsifying that these distortions are simply due to a ‘factual’ status-size heuristic (thus, the association is the same for high-status individuals overall) by comparing judgments of in-group versus out-group leaders. Furthermore, we will extend the literature by examining the prestige effects of performance on these distortions, and by not only using height measurements, but also strength measurements (perceived strength and muscularity). We will also include weight measurements to test that these effects are indeed related to fighting ability and not to body size in general. Although previous research has shown that weight is related to perceived fighting ability for mixed-martial-arts fighters [68], we expect that this is due to weight being related to muscle mass, and does not translate to politicians, who are not trained fighters. Next, we will investigate whether there are gender effects in formidability perceptions, and whether these effects are due to using one’s own height as an anchor. Lastly, we use a more critical design in which we also look at time differences within participants in formidability perceptions of leaders (within-subject design).

## Method

All the research reported in this document was approved by the Scientific and Ethical Review Committee (VCWE) of the faculty of Behavioural and Movement Sciences at Vrije Universiteit Amsterdam. Data were gathered online via Qualtrics software (Qualtrics, Provo, UT). Before starting the study, participants were briefed on the study and had to click ‘yes’ or ‘no’ for their consent. The study commenced upon agreement, or was ended when ‘no’ was clicked. At the end of the study, participants could opt for information via email when the results of the study were known. They were also given contact details of the primary investigators to be used for questions or complaints.

### Participants

Participants were recruited via a voting advice website where Dutch people can test their political preference by answering to a series of statements (www.stemwijzer.nl). The website had a short invitation to participate in a study about the Dutch general elections of 2012, with a link to the study website. For the first part of the study, a total of 2,013 non-random, self-selected participants agreed to participate. Data of two participants were removed because they made extremely unrealistic judgments (e.g., weight estimates of 900 kilograms). Of the remaining 2,011 participants (1,026 males, 985 females; *M*_age_ = 46.28 years, *SD* = 16.30 years), 99.7% had suffrage for the general Dutch elections, and 94.8% were planning on voting for the 2012 elections. We also asked participants: “Which party would you vote for, if elections were held today?” Of the 1,954 participants who answered this question, 73.4% were planning to vote for one of the six parties whose leaders we included in the study. Of those participants, the majority (66.0%) were planning to vote for VVD, PvdA, or SP (Table 1).

**Table 1:** Percentages of (intended) votes for each political party in the 2012 Dutch general elections.

Political party	% intended votes Part 1 [*N* = 1.954]	% actual votes Part 2 [*N* = 269]	% votes in elections 2012	% votes in elections 2010
PvdA*	16.7	29.5	24.84	19.63
VVD*	16.1	17.4	26.58	20.49
SP*	15.7	11.8	9.65	9.82
D66*	11.7	14.0	8.03	6.95
PVV*	9.5	4.3	10.08	15.45
**GroenLinks**	5.8	6.8	2.33	6.67
CDA*	3.7	4.3	8.51	13.61
**Christen Unie**	2.8	2.8	3.13	3.24
**Partij voor de Dieren**	1.8	1.9	1.93	1.30
**SGP**	1.5	1.9	2.09	1.74
**Different**	6.0	2.8	2.83	1.10
**Chose not to answer**	5.8	2.5	-	-

*Parties whose leaders were estimated in the current study.

For the second part of the study, 599 participants (29.8% response rate) agreed to participate. Participants were asked to create a unique code, which would allow us to anonymously match data from parts one and two. Unfortunately, a lot of the participants created different codes for the separate parts of the study, or did not enter any code at all. We were able to successfully match data of parts one and two for 322 of these participants (16.0% of Wave1; 161 males, 161 females; *M*_age_ = 46.54, *SD* = 15.85). Again, 99.7% had suffrage for the general Dutch elections (two participants chose to not answer this question), and 99.1% (*N* = 319) had actually voted in the elections. Of the 269 participants who disclosed which party they voted for, most (60.9%) voted for PvdA, VVD, or D66 (Table 1).

As compensation for taking part in the study the participants were entered into a raffle (once if they only completed the first part, twice if they also completed the second part of the study) to win one of ten vouchers with a monetary value of €20 each.

### Materials and procedure

**Fig 1:**
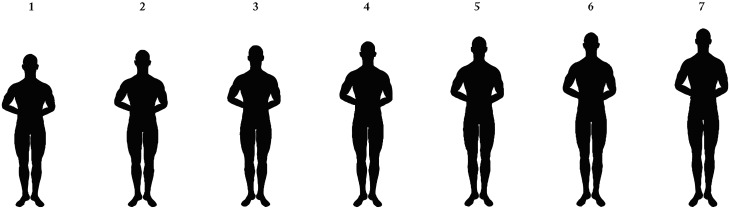
Height silhouettes used for measuring estimated height.

**Fig 2:**
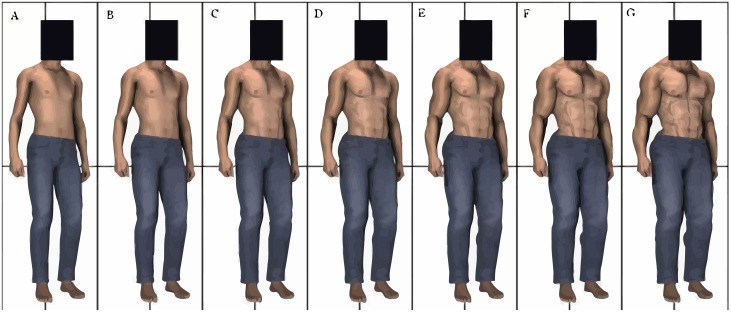
Muscularity silhouettes used for measuring estimated strength (from Holbrook & Fessler, 2013).

After some sociodemographic questions, participants were randomly assigned to one of the party leaders (all male) of the six largest participating political parties. These were Sybrand van Haersma Buma (CDA), Alexander Pechtold (D66), Emile Roemer (SP), Mark Rutte (VVD), Diederik Samsom (PvdA), and Geert Wilders (PVV; for (perceived) heights, weights, and strengths of the individual party leaders, see Table 2). We determined whether participants were assessing an in-group or out-group party leader by comparing their voting intentions (which party they were planning to vote on) to the party leader randomly assigned to them. Table 3 shows the number of participants in the in-group and out-group conditions for each estimated political leader (data from Wave1). Participants were presented with a small, standardized black and white headshot (134 px by 163 px) of the party leader and the following text: “*– Name leader-*, *- Name party-*. *We would like to know about your first impressions of – name leader-*. *Please answer the following questions about him; please remember these are estimates*.*”* Next, each participant gave 11 estimations in total of their liking of, and perceptions of formidability, age, and political skills of the assigned party leader. Liking was measured with one item: “*How do you perceive –name party leader-*?” with sliders that indicated liking from 0 (*very negatively*) to 100 (*very positively*). Formidability was estimated through height and strength perceptions of the party leaders. Height was estimated (1) in centimeters, (2) with sliders that indicated height from 0 (*very short)* to 100 (*very tall*), and (3) with seven male silhouettes that ranged from short to tall (Fig 1). Strength was estimated (1) with sliders that indicated physical strength from 0 (*very weak*) to 100 (*very strong*), and (2) with seven male body images that ranged from not so muscular to very muscular (from Holbrook & Fessler [53]; Fig 2). Weight was estimated (1) in kilograms and (2) with sliders that indicated weight from 0 (*very light*) to 100 (*very heavy)*. An overview of all the height, weight, and strength measures can be found in Table 4. Age was estimated (1) in years and (2) with sliders that indicated age from 0 (*very young)* to 100 (*very old*). Finally, political skills were estimated with one item: sliders that indicated political skills from 0 (*very bad)* to 100 (*very good*). Each participant answered questions on only one randomly selected party leader (between-subject design).

**Table 2:** Estimated and actual heights and estimated weights and strength.

Party leader	Actual height	Perceived height before elections	Perceived height after elections	Perceived weight before elections	Perceived weight after elections	Perceived strength before elections	Perceived strength after elections
**Haersma Buma**	180	181.52 (*SD* = 5.00)	180.59 (*SD* = 6.79)	82.83 (*SD* = 6.34)	82.79 (*SD* = 6.11)	54.11 (*SD* = 8.14)	53.67 (*SD* = 15.58)
**Pechtold**	186	178.23 (*SD* = 5.10)	178.85 (*SD* = 5.78)	79.18 (*SD* = 5.78)	79.63 (*SD* = 5.08)	54.77 (*SD* = 18.57)	59.07 (*SD* = 16.32)
**Roemer**	190	180.74 (*SD* = 5.85)	181.88 (*SD* = 7.08)	91.26 (*SD* = 9.26)	91.86 (*SD* = 9.23)	59.74 (*SD* = 18.51)	65.02 (*SD* = 13.73)
**Rutte**	191	181.91 (*SD* = 5.63)	180.92 (*SD* = 7.02)	79.12 (*SD* = 6.10)	79.04 (*SD* = 6.60)	56.37 (*SD* = 18.19)	60.71 (*SD* = 15.04)
**Samsom**	175	178.81 (*SD* = 5.87)	177.66 (*SD* = 5.40)	78.35 (*SD* = 6.54)	77.04 (*SD* = 5.03)	61.91 (*SD* = 16.29)	67.11 (*SD* = 13.83)
**Wilders**	195	181.10 (*SD* = 5.73)	182.00 (*SD* = 5.89)	82.21 (*SD* = 6.58)	84.22 (*SD* = 7.29)	58.00 (*SD* = 19.28)	58.06 (*SD* = 15.60)

Notes: Heights in centimeters, weights in kilograms, strength in slider measurement 0–100.

**Table 3:** Number of participants per estimated politician, per condition.

Politician	In-group *n*	Out-group *n*	Chose not to answer *n*	Total *n*
Rutte	62	310	36	408
Roemer	54	259	32	345
Samsom	45	237	26	308
Wilders	33	258	29	320
Pechtold	42	239	24	305
Buma	11	288	26	325
Total	247	1591	173	2011

**Table 4:** Descriptive statistics for the seven measures of height, strength, and weight.

Measure	Wave 1		Wave 2	
	*Mean*	*SD*	*Mean*	*SD*
Height cm	180.48	5.71	180.36	6.49
Height slider	66.08	14.73	65.75	13.77
Height silhouettes	4.19	1.41	4.12	1.31
Strength slider	57.45	18.37	60.33	15.63
Strength silhouettes	1.98	1.24	2.08	1.14
Weight kg	82.19	8.22	82.66	8.25
Weight slider	67.22	16.01	66.30	14.56

We contacted the offices of all of the party leaders with requests for their actual heights. Only Rutte and Pechtold responded to our request (Samsom indicated he did not wish to cooperate, the others did not reply). We therefore made educated guesses for the heights of the other four leaders with the help of internet sources and footage, by using multiple sources on the internet stating the party leaders’ height, and using footage to assess height differences between the party leaders who responded and the others. This resulted in the ‘actual height’ column in Table 2. As not having accurate measures of the party leaders’ heights is a limitation, we have included analyses on the accuracy of height estimations in the Electronic Supplementary Materials, but will not discuss them in the main text.

Finally, we asked for self-reported height and weight of the participants, their involvement and interest in the current elections, their voting intentions, their past voting behavior, their expectations about election outcomes, and whether they were a member of a political party. Next, they were debriefed and thanked for their participation.

After the elections, participants who had indicated that they would like to participate in a second part of the study were emailed a link to an online survey. Upon visiting the study website, participants were first presented with an informed consent form and after giving their consent, were taken to the actual study. Next, they completed the same 11 questions on the same party leader as they did in the first part of the study. We also asked them whether they voted, and if so, for which party, how satisfied they were with their choice, and who they thought the biggest winning and losing parties were. After this, they were debriefed and thanked for their participation, and could take part in the raffle if they wished so.

### Analyses

We expected that formidability estimations would be larger for in-group leaders (leaders from a political party that someone would vote for) than for out-group leaders (leaders from a political party that someone would *not* vote for). We also expected that when leaders are seen as more prestigious, they would also be seen as more physically formidable, and that men would make larger overestimations than women would make. We controlled for liking and estimated age, because this could have effects on leadership perceptions (e.g., [69]). Lastly, we expected that after the elections, winners would be estimated as more formidable, while losers would be estimated as less formidable, compared to before the elections.

Given that in this study participants only made judgments for a single party leader, we conducted analyses of covariance (ANCOVA’s) and linear regression analyses for analyzing the first part of the study. For the second part of the analyses (the winner-loser effects) the data are nested: multiple ratings in time made by individuals, and therefore we opted for repeated measures mixed models in SPSS version 21’s Mixed procedure [70] with restricted maximum likelihood estimators (REML). As a robustness check, we applied a bootstrapping procedure (1,000 resamples, 95% CI Bias Corrected and Accelerated) for each of our main analyses. All estimation variables were centered by subtracting the mean of all scores for a specific variable from the individual score of that variable (*grand mean centering*). Self-reported height and weight of the participants were transformed into *z*-scores separately for men and women. To be able to take into account as many data points as possible, we chose to also analyze data of participants who had some missing values, rather than opt for ‘listwise deletion’. Due to this, the degrees of freedom will differ between analyses.

## Results

Table 5 shows the correlation table (bivariate correlations, two-tailed) for all the party leader measurements. As the two age measurements were moderately to highly correlated with each other (*r*(2007) = .586, *p* < .001), we decided to transform these values into one variable (reliability of the two measurements α = .690). As Table 5 shows, the different height, weight, and strength measurements did not all correlate strongly with each other (*r*’s ranging from .035 to .507). Therefore, and in order to compare outcomes of different means of measurement, we decided to run separate analyses for all the height, weight, and strength measurements. We present our results in four different sections: first, we discuss the results concerning in-group leaders vs. out-group leaders (P1 –P3); second, we discuss the results concerning prestige (P4 –P6); third, we discuss the results concerning winner and loser effects (P7 –P9); and fourth, we discuss the results concerning perceiver sex (P10 –P11). Lastly, we discuss exploratory analyses examining whether perceiver sex differences can be explained by anchoring or sensitivity to opponents’ physical formidability. All analyses are conducted with our largest sample of Wave1 (*N* = 2,011), except for the repeated measures analyses concerning the winner/loser effects (Part 3, P7 –P9), which are conducted with the smaller sample of participants who completed both waves (*N* = 322).

**Table 5:** Correlation table.

	**Height slider**	**Height CM**	**Height Silh.**	**Strength Slider**	**Strength Silh.**	**Weight Slider**	**Weight KG**	**Pol. Skills**	**Liking**	**Age Slider**	**Age Years**
**Height** slider	1	.495**	.455**	.334**	.116**	.338**	.136**	.209**	.188**	.065**	.016
**Height** CM		1	.507**	.146**	.052*	.123**	.344**	.123**	.101**	.039	-.032
**Height** Silh.			1	.211**	.044*	.076**	.081**	.172**	.162**	-.021	-.061**
**Strength** Slider				1	.285**	.176**	.035	.514**	.517**	-.004	-.048*
**Strength** Silh.					1	.157**	.125**	.115**	.180**	.028	.097**
**Weight** Slider						1	.337**	.022	.059**	.156**	.171**
**Weight** KG							1	-.077**	-.011	.220**	.218**
**Pol. Skills**								1	.665**	-.016	-.084**
**Liking**									1	-.026	-.032
**Age** Slider										1	.586**
**Age** Years											1

*p < .05.

**p < .01.

### 1. In-group leaders vs. out-group leaders

To test whether in-group leaders were perceived as more formidable than out-group leaders, we conducted linear regression analyses for each of the formidability measures. When prestige significantly predicted the independent and dependent variable, we conducted a Sobel test [71] in order to test whether prestige acted as a mediator. Table 6 shows the means and standard deviations of all the height, strength and weight measures for in-group and out-group leaders (data from Wave1).

**Table 6:** Perceived height, strength, and weight for in-group leaders and out-group leaders.

Measure	In-group leaders		Out-group leaders	
	*M*	*SD*	*M*	*SD*
Height cm	181.26	5.73	180.42	5.76
Height slider	68.58	14.56	66.02	14.42
Height silhouettes	4.41	1.33	4.17	1.44
Strength slider	67.75	15.58	55.61	17.87
Strength silhouettes	2.20	1.39	1.95	1.21
Weight kg	82.91	7.77	82.13	8.27
Weight slider	68.92	16.35	67.08	16.20

#### Height

**Fig 3:**
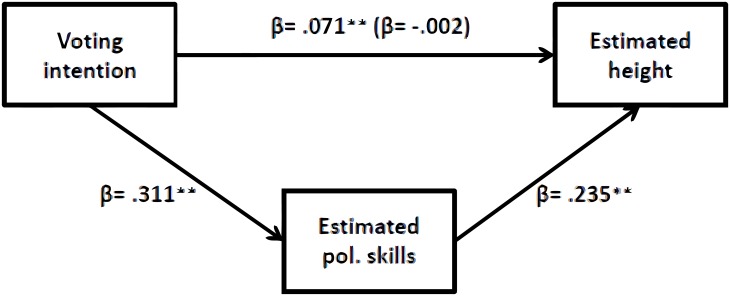
Effect of voting intention on estimated height is mediated by estimated political skills.

Our first prediction (P1) stated that in-group leaders would be estimated as taller compared to out-group leaders. Three linear regression analyses showed that in-group party leaders were estimated as taller than out-group party leaders in all three height measurements (Table 7). This effect was fully mediated by estimated political skills; cm height measurements: *z* = 5.20, *SE* = .14; *p* < .001; slider height measurements: *z* = 7.16, *SE* = .39; *p* < .001; height silhouette measurements: *z* = 5.77, *SE* = .04; *p* < .001. Fig 3 illustrates the results on the slider height measurements.

**Table 7:** Linear regression of height on in-group leaders vs. out-group leaders.

	Δ*R*^2^	*B*	*SE B*	*ß*	*p*	Bootstrap *p*	Bootstrap 95% CI
**Height cm**							
Step 1	.00						
Constant		-.09	.16		.594	.619	-.41, .22
Group		1.01	.39	.07	.010	.008	.22, 1.79
Step 2	.02						
Constant		.01	.16		.952	.955	-.31, .31
Group		.30	.41	.02	.458	.468	-.54, 1.14
Prestige		.04	.01	.15	<.001	.001	.03, .05
**Height slider**							
Step 1	.01						
Constant		-.07	.41		.872	.860	-.92, .81
Group		2.71	.99	.07	.006	.011	.66, 4.75
Step 2	.05						
Constant		.31	.40		.442	.408	-.47, 1.10
Group		-.08	1.02	-.00	.936	.950	-2.03, 1.78
Prestige		.16	.02	.24	<.001	.001	.12, .20
**Height silh.**							
Step 1	.01						
Constant		-.02	.04		.619	.640	-.10, .06
Group		.26	.10	.07	.008	.003	.08, .44
Step 2	.04						
Constant		.01	.04		.767	.774	-.06, .09
Group		.02	.10	.01	.830	.813	-.16, .22
Prestige		.01	.00	.20	<.001	.001	.01, .02

#### Strength

**Fig 4:**
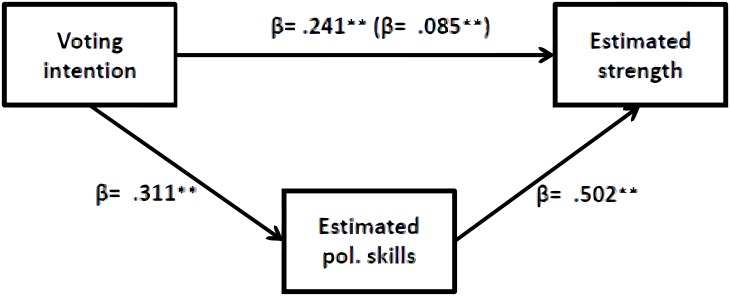
Effect of voting intention on estimated strength is partly mediated by estimated political skills.

Our second prediction (P2) stated that in-group leaders would be estimated as stronger compared to out-group leaders. In line with our predictions, two linear regression analyses showed that in-group party leaders were estimated as stronger than out-group party leaders in both strength measurements (Table 8). The effect of party leader group on strength measured with sliders was partially mediated by prestige: *z* = 10.91, *SE* = .68; *p* < .001 (Fig 4), and the effect of party leader group on strength measured with silhouettes was fully mediated by prestige: *z* = 3.81, *SE* = .04; *p* < .001).

**Table 8:** Linear regression of strength on in-group leaders vs. out-group leaders.

	Δ*R*^2^	*B*	*SE B*	*ß*	*p*	Bootstrap *p*	Bootstrap 95% CI
**Strength slider**							
Step 1	.06						
Constant		-.75	.50		<.001	.002	-2.75, -.85
Group		11.53	1.21	.24	<.001	.001	9.45, 13.61
Step 2	.23						
Constant		-.75	.43		.083	.081	-1.65, .06
Group		4.07	1.11	.09	<.001	.001	2.19, 6.01
Prestige		.42	.02	.50	<.001	.001	.37, .47
**Strength silh.**							
Step 1	.01						
Constant		-.03	.03		.402	.393	-.09, .04
Group		.23	.08	.07	.006	.013	.06, .42
Step 2	.02						
Constant		-.01	.03		.771	.744	-.07, .06
Group		.09	.09	.03	.307	.311	-.08, .29
Prestige		.01	.00	.14	<.001	.001	.01, .01

#### Weight

Our third prediction (P3) stated that there would be no difference in weight estimations between in-group and out-group leaders. In line with our predictions, two linear regression analyses showed no significant effects of in-group vs. out-group for the weight in kilograms and weight in slider measurements (Table 9).

**Table 9:** Linear regression of weight on in-group leaders vs. out-group leaders.

	Δ*R*^2^	*B*	*SE B*	*ß*	*p*	Bootstrap *p*	Bootstrap 95% CI
**Weight kg**							
Step 1	.00						
Constant		-.06	.20		.751	.744	-.46, .29
Group		.76	.48	.04	.116	.103	-.04, 1.68
Step 2	.00						
Constant		-.07	.20		.712	.705	-.46, .27
Group		.84	.51	.05	.098	.076	.01, 1.78
Prestige		-.01	.01	-.01	.604	.644	-.03, .01
**Weight slider**							
Step 1	.00						
Constant		-.13	.44		.766	.754	-.92, .69
Group		1.74	1.07	.04	.104	.110	-.50, 3.96
Step 2	.00						
Constant		-.07	.44		.878	.867	-.86, .75
Group		1.27	1.13	.03	.259	.272	-.93, 3.55
Prestige		.03	.02	.04	.181	.247	-.02, .07

### 2. Prestige

We expected that estimated prestige would have a positive association with estimated height and strength, but not with estimated weight. We discuss the effects of interest to our hypotheses (political skills, participant sex, and participant height/weight) here. For the effects of the control variables estimated age and liking, please see the ESM.

#### Height

**Fig 5:**
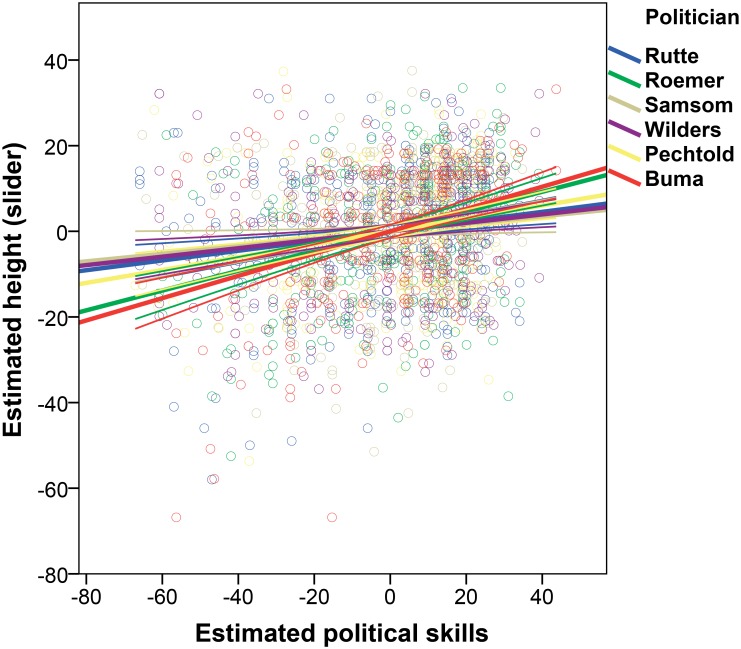
Estimated height by estimated political skills for the individual party leaders. Notes: Rutte: *N* = 408, *R*^2^ = .030; Roemer: *N* = 345, *R*^2^ = .106; Samsom: *N* = 308, *R*^2^ = .013; Wilders: *N* = 320, *R*^2^ = .029; Pechtold: *N* = 305, *R*^2^ = .054; van Haersma Buma: *N* = 325, *R*^2^ = .124.

Our fourth prediction (P4) stated that perceived prestige of leaders would have a positive association with perceived height of leaders. We conducted three analyses of covariance (ANCOVAs) with estimated height (1) in centimeters, (2) in sliders, and (3) in silhouettes of the six party leaders as dependent variable, and participant sex, participant height, and estimated political skills, estimated ages, and liking of the six leaders as independent variables (overall models: (1) *F*(5, 1953) = 11.317, *p* < .001, *R*^2^ = .028; (2) *F*(5, 1963) = 27.288, *p* < .001, *R*^2^ = .065; (3) *F*(5, 1963) = 20.014, *p* < .001, *R*^2^ = .049). In line with our expectations, estimated political skills had a significant effect on estimated height for all three height measures: when party leaders were estimated to have better political skills, they were estimated to be taller: (1) *t*(1959) = 2.901, *p* = .004, η_p_^2^ = .004, 95% CI [.01, .04]; bootstrap: *p* = .015, 95% CI [.00, .04]; (2) *t*(1969) = 4.056, *p* < .001, η_p_^2^ = .008, 95% CI [.04, .12]; bootstrap: *p* = .002, 95% CI [.039, .130]; (3) *t*(1969) = 3.249, *p* = .001, η_p_^2^ = .005, 95% CI [.00, .01]; bootstrap: *p* = .006, 95% CI [.00, .01]. Fig 5 illustrates the estimated height via sliders by estimated political skills for all six party leaders.

#### Strength

**Fig 6:**
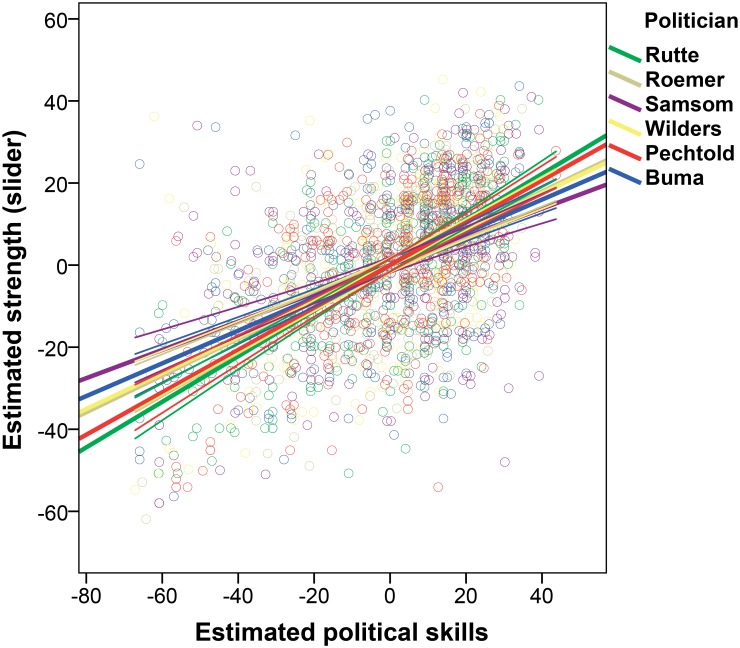
Estimated strength by estimated political skills for the individual party leaders. Notes: Rutte: *N* = 408, *R*^2^ = .223; Roemer: *N* = 345, *R*^2^ = .410; Samsom: *N* = 308, *R*^2^ = .273; Wilders: *N* = 320, *R*^2^ = .198; Pechtold: *N* = 305, *R*^2^ = .254; van Haersma Buma: *N* = 325, *R*^2^ = .338.

Our fifth prediction (P5) stated that perceived prestige of leaders would have a positive association with perceived strength of leaders. We conducted two analyses of covariance (ANCOVAs) with estimated strength (1) via sliders and (2) via silhouettes of the six party leaders as dependent variable, and participant sex, estimated political skills, estimated ages, and liking of the six leaders as independent variables (overall models: (1) *F*(4, 2006) = 260.062, *p* < .001, *R*^2^ = .341; (2) *F*(4, 2006) = 16.862, *p* < .001, *R*^2^ = .033). In line with our expectations, estimated political skills had a significant effect on estimated strength in the sliders measurement: when party leaders were estimated as more prestigious, they were estimated to be stronger: (1) *t*(2011) = 11.117, *p* < .001, η_p_^2^ = .058, 95% CI [.20, .28]; bootstrap: *p* = .001, 95% CI [.19, .29]. However, against our expectations, we did not find a significant effect of prestige on estimated strength in the silhouettes measurement: (2) *t*(2011) = 1.045, *p* = .296. Fig 6 illustrates the estimated strength via sliders by estimated political skills for all six party leaders.

#### Weight

Our sixth prediction stated that perceived prestige of leaders would not be significantly associated with perceived weight of leaders. We conducted two analyses of covariance (ANCOVAs) with estimated weight (1) in kilograms, and (2) via sliders of the six party leaders as dependent variable, and participant sex, participant weight, and estimated political skills, estimated ages, and liking of the six leaders as independent variables (overall models: (1) *F*(5, 1947) = 5.559, *p* < .001, *R*^2^ = .014; *F*(5, 1952) = 3.845, *p* = .002, *R*^2^ = .010). In line with our expectations, estimated political skills did not have a significant effect on either of the estimated weight measurements: (1) *t*(1953) = -.360, *p* = .719; (2) *t*(1958) = .138, *p* = .890.

### 3. Winner/loser effects

We expected that the party leader(s) who won the elections would be estimated as more formidable after the elections compared to before the elections, and that the party leaders who lost the elections would be estimated as less formidable after the elections compared to before the elections. Table 1 shows the percentages of intended and actual votes given to each party in this study, and the percentages of actual votes in the last and current elections. The 2012 Dutch general elections campaign started out as a competition between Rutte and Roemer over the largest number of votes (and thus the prime-ministership), but quickly turned into a battle between Rutte and Samsom [72–74]. Rutte’s party eventually gained 10 seats in parliament, becoming the largest party, and even though Samsom gained 8 seats, he lost the prime-ministership. Objectively, the ‘biggest losers’ were Wilders (-9 seats) and van Haersma-Buma (-8 seats). In spite of the strong positive expectations early in the elections, Roemer’s number of seats remained equal compared to before the elections. Finally, Pechtold gained two seats (all these data are from www.rijksoverheid.nl). The participants in our study actually viewed Samsom as the greatest winner (49.1%), with Rutte following closely as the second greatest winner (48.2%). They viewed Wilders as the greatest loser (36.8%), followed closely by Jolande Sap (GroenLinks, not estimated in this study; 36.2%).

For the winner/loser effects analyses, we centered all the leader estimation variables with their mean over both parts of the study (*grand mean centering*). We then conducted two repeated measures mixed model analyses for each of the party leaders separately: an unstructured covariance model (UNR) allowing for different variances between measurements in time (model 1), and an autoregressive model (AR1) where the variance is the same for each measurement in time (model 2; [75]). We did this for each of the seven height, strength, and weight estimations. The units of analysis were time (before elections/after elections), group (in-group: participant voted for the party of the judged leader/ out-group: participant did not vote for the party of the judged leader) and height, weight, liking, age and performance estimations of the party leaders made by the participants.

#### Height

Our seventh prediction (P7) stated that winners would be estimated as taller compared to losers. For each party leader, we conducted two repeated measures mixed models with estimated height (1) in centimeters, (2) via sliders, and (3) via silhouettes of the party leaders as dependent variables and time, group, liking, estimated age, estimated political skills, and the interaction between time and group as independent variables. Overall, the first model (unstructured) was comparatively the best fitting model for (1), while the second model (autoregressive) was comparatively the best fitting model for (2) and (3), and therefore these models are reported in Tables 10–12. We found two (out of three) significant robust effects of time on estimated height for Samsom: (1) *t*(42.320) = 2.129, *p* = .039, 95% CI [.13, 4.80]; (2) *t*(42.895) = 1.747, *p* = .088, 95% CI [-.77, 10.71]; (3) *t*(43.925) = 2.196, *p* = .033, 95% CI [.05, 1.24]. Samsom was estimated taller before the elections compared to after the elections when estimating in centimeters (before: *M* = 1.46, *SD* = 1.01; after: *M* = -.97, *SD* = .81) or via the height silhouettes (before: *M* = .44, *SD* = .20; after: *M* = -.33, *SD* = .20). Interestingly, we also found a significant effect of time on estimated height via silhouettes for Wilders: *t*(50.080) = -2.643, *p* = .011, 95% CI [-.76, -.10] and van Haersma Buma: *t*(53.056) = 2.375, *p* = .021, 95% CI [.07, .80]. Van Haersma Buma was estimated taller before the elections (*M* = .25, *SD* = .18) compared to after the elections (*M* = -.19, *SD* = .18). Wilders was estimated shorter before the elections (*M* = -.24, *SD* = .19) compared to after the elections (*M* = .17, *SD* = .19).

**Table 10:** Repeated measures mixed models with estimated height in centimeters.

Parameter	Model fit		Time		Group		Liking		Estimated age		Est. pol. skills		Time x Group	
Party leader	Δ *AIC*	Δ *BIC*	*b*	*SE*	*b*	*SE*	*b*	*SE*	*b*	*SE*	*b*	*SE*	*b*	*SE*
Rutte	51.322	52.845	-3.26	4.07	4.40	2.70	-.06	.06	.16	.11	.12	.08	4.80	8.81
Roemer	0.083	-2.57	-.26	2.24	-2.30	3.28	-.02	.04	-.01	.06	.00	.04	-.26	2.24
Samsom	0.95	-1.444	2.46*	1.16	.75	1.89	-.02	.05	.06	.06	.06	.05	-.10	1.96
Wilders	-1.854	-4.428	-1.44	.78	.62	6.49	.01	.03	.02	.06	.06	.03	-.82	5.52
Pechtold	74.782	72.091	-4.25	3.33	.83	1.98	-.07	.07	.09	.08	.11	.09	5.16	7.31
Haersma Buma	3.989	1.298	1.24	.67	-.09	3.53	.02	.04	.01	.05	.02	.04	-.60	2.56

Notes: Δ AIC / Δ BIC: difference with the autoregressive model (positive number is better fit).

*p < .05.

**Table 11:** Repeated measures mixed models with estimated height in sliders.

Parameter	Model fit		Time		Group		Liking		Estimated age		Est. pol. skills		Time x Group	
Party leader	Δ *AIC*	Δ *BIC*	*b*	*SE*	*b*	*SE*	*b*	*SE*	*b*	*SE*	*b*	*SE*	*b*	*SE*
Rutte	1.994	4.471	2.11	1.88	3.86	4.51	.03	.08	.22	.15	.26**	.10	-2.5	4.02
Roemer	1.989	4.643	-2.67	1.79	.88	5.98	-.10	.08	.04	.13	.16	.10	-.04	5.22
Samsom	1.424	3.819	4.97	2.85	3.14	4.75	.07	.11	.17	.15	.15	.12	-.23	4.84
Wilders	1.879	4.453	-1.74	1.86	6.93	13.21	.10	.07	.02	.12	.12	.07	-12.95	13.19
Pechtold	1.734	4.426	-.14	1.93	-.95	4.40	.21*	.10	.18	.13	-.02	.12	5.25	4.19
Haersma Buma	1.065	3.756	.18	1.57	-.13	6.03	.22*	.10	.12	.12	.00	.10	-1.53	6.03

Notes: Δ AIC / Δ BIC: difference with the unstructured model (positive number is better fit).

*p < .05.

**p < .01.

**Table 12:** Repeated measures mixed models with estimated height in silhouettes.

Parameter	Model fit		Time		Group		Liking		Estimated age		Est. pol. skills		Time x Group	
Party leader	Δ *AIC*	Δ *BIC*	*b*	*SE*	*b*	*SE*	*b*	*SE*	*b*	*SE*	*b*	*SE*	*b*	*SE*
Rutte	1.700	4.178	.17	.17	.06	.44	.01	.01	.01	.01	.00	.01	.17	.35
Roemer	1.712	4.366	-.14	.19	-.32	.63	.01	.01	.00	.01	.00	.01	-.05	.57
Samsom	1.957	4.351	.65*	.29	-.14	.45	.01	.01	-.00	.01	.01	.01	.33	.50
Wilders	1.946	4.521	-.43*	.16	.10	1.43	-.00	.01	-.02	.01	.02**	.01	.91	1.16
Pechtold	1.998	4.689	.03	.17	.00	.37	.00	.01	-.00	.01	.01	.01	-.02	.36
Haersma Buma	1.738	4.429	.43*	.18	-.44	.70	.01	.01	.00	.01	.00	.01	.05	.70

Notes: Δ AIC / Δ BIC: difference with the unstructured model (positive number is better fit)

*p < .05,

**p < .01.

#### Strength

Our eighth prediction (P8) stated that winners would be estimated as stronger compared to losers. For each party leader, we conducted two repeated measures mixed models with estimated strength (1) via sliders, and (2) via silhouettes of the party leaders as dependent variables and time, group, liking, estimated age, estimated political skills, and the interaction between time and group as independent variables. Overall, the second model (autoregressive) was comparatively the best fitting model and these models are therefore reported in Tables 13 and 14. Surprisingly, we found contrasting effects of time on estimated strength for van Haersma Buma: (1) *t*(53.484) = 2.097, *p* = .041, 95% CI [.17, 7.68]; (2) *t*(53.064) = -2.684, *p* = .010, 95% CI [-.52, -.08]. When estimating via sliders, Van Haersma Buma was estimated stronger before the elections (*M* = 2.64, *SD* = 1.68) compared to after the elections (*M* = -1.08, *SD* = 1.68). However, when estimating via silhouettes, Van Haersma Buma was estimated less strong before the elections (*M* = -.19, *SD* = .14) compared to after the elections (*M* = .07, *SD* = .14). Furthermore, we found a significant effect of time on estimated strength via silhouettes for Roemer (*t*(55.512) = -2.207, *p* = .032, 95% CI [-.85, -.04]) and Samsom (*t*(44.193) = -2.387, *p* = .021, 95% CI [-.55, -.05]). Against our expectations, Roemer and Samsom were estimated less strong before the elections (Roemer: *M* = -.14, *SD* = .20; Samsom: *M* = -.15, *SD* = .12) compared to after the elections (Roemer: *M* = .22, *SD* = .20; Samsom: *M* = .15, *SD* = .12).

**Table 13:** Repeated measures mixed models with estimated strength in sliders.

Parameter	Model fit		Time		Group		Liking		Estimated age		Est. pol. skills		Time x Group	
Party leader	Δ *AIC*	Δ *BIC*	*b*	*SE*	*b*	*SE*	*b*	*SE*	*b*	*SE*	*b*	*SE*	*b*	*SE*
Rutte	1.805	4.282	-3.41	2.03	.66	4.74	.31**	.09	-.07	.16	.33**	.11	-4.12	4.85
Roemer	1.396	4.049	-1.45	1.84	6.61	5.45	.19*	.08	.12	.13	.18	.09	.36	5.40
Samsom	.341	2.735	-.07	2.27	5.15	4.36	.07	.10	-.26*	.13	.28**	.11	-1.76	3.84
Wilders	1.998	4.573	-2.75	2.39	-.28	15.09	.26**	.08	.08	.14	.15	.08	-6.48	17.01
Pechtold	1.879	4.570	-3.25	1.87	.65	4.80	.25*	.10	.34*	.13	.23	.12	4.29	4.07
Haersma Buma	-8.805	-0.114	3.93*	1.87	2.96	6.99	.23*	.10	.07	.12	.20	.11	-2.98	7.17

Notes: Δ AIC / Δ BIC: difference with the unstructured model (positive number is better fit)

*p < .05.

**p < .01.

**Table 14:** Repeated measures mixed models with estimated strength in silhouettes.

Parameter	Model fit		Time		Group		Liking		Estimated age		Est. pol. skills		Time x Group	
Party leader	Δ *AIC*	Δ *BIC*	*b*	*SE*	*b*	*SE*	*b*	*SE*	*b*	*SE*	*b*	*SE*	*b*	*SE*
Rutte	2.000	4.477	.03	.11	-.33	.28	.01*	.00	.01	.01	-.01	.01	.13	.23
Roemer	.688	3.342	-.45*	.20	.51	.66	-.00	.01	.02	.02	.01	.01	.82	.59
Samsom	1.610	4.004	-.30*	.13	.03	.28	.00	.01	-.02*	.01	.01	.01	-.01	.28
Wilders	1.681	4.255	-.22	.15	-1.65	1.14	.01	.01	-.01	.01	.00	.01	.23	1.03
Pechtold	1.309	4.000	.08	.10	-.19	.24	.01*	.01	-.00	.01	-.01	.01	-.16	.21
Haersma Buma	1.347	4.039	-.30*	.11	-.05	.43	.01	.01	.00	.01	-.00	.01	.56	.43

Notes: Δ AIC / Δ BIC: difference with the unstructured model (positive number is better fit).

*p < .05.

#### Weight

Our ninth prediction (P9) stated that there would be no difference in weight estimations between winners and losers. For each party leader, we conducted two repeated measures mixed models with estimated weight (1) in kilograms, and (2) via sliders of the party leaders as dependent variables and time, group, liking, estimated age, estimated political skills, and the interaction between time and group as independent variables. Overall, the second model (autoregressive) was comparatively the best fitting model and is therefore reported in Tables 15 and 16. We found a significant effect of time on estimated weight in kilograms for Samsom: (1) *t*(42.791) = 2.898, *p* = .006, 95% CI [.98, 5.49]. Samsom was estimated heavier before the elections (*M* = 1.45, *SD* = .83) compared to after the elections (*M* = -1.46, *SD* = .83). We also found a marginally significant effect of time on estimated weight in kilograms for Roemer: (1) *t*(54.389) = -1.960, *p* = .055, 95% CI [-3.99, .04]. However, the confidence interval suggests that this is not a robust effect. We found no significant effects of time on estimated weight in sliders of the party leaders.

**Table 15:** Repeated measures mixed models with estimated weight in kilograms.

Parameter	Model fit		Time		Group		Liking		Estimated age		Est. pol. skills		Time x Group	
Party leader	Δ *AIC*	Δ *BIC*	*b*	*SE*	*b*	*SE*	*b*	*SE*	*b*	*SE*	*b*	*SE*	*b*	*SE*
Rutte	-.485	1.992	-.72	.92	3.74	2.30	-.04	.04	.11	.07	.00	.05	1.68	1.97
Roemer	1.821	4.475	-1.97	1.01	-2.85	4.08	.01	.05	.05	.08	-.05	.06	1.82	2.91
Samsom	1.710	4.104	3.24**	1.12	3.19	1.91	-.06	.04	.04	.06	.07	.05	-.96	1.90
Wilders	1.452	4.027	-.41	.88	3.74	6.76	.01	.04	.12	.06	-.02	.03	-2.53	6.23
Pechtold	1.986	4.677	-.57	.62	-1.33	1.74	-.01	.04	.08	.05	.08	.04	2.68	1.34
Haersma Buma	1.999	4.690	.19	.69	-1.62	3.15	-.01	.04	.12*	.05	-.01	.04	.68	2.64

Notes: Δ AIC / Δ BIC: difference with the unstructured model (positive number is better fit).

*p < .05.

**p < .01

**Table 16:** Repeated measures mixed models with estimated weight in sliders.

Parameter	Model fit		Time		Group		Liking		Estimated age		Est. pol. skills		Time x Group	
Party leader	Δ *AIC*	Δ *BIC*	*b*	*SE*	*b*	*SE*	*b*	*SE*	*b*	*SE*	*b*	*SE*	*b*	*SE*
Rutte	1.989	4.466	4.09	2.79	6.98	5.63	-.04	.10	.15	.19	.22	.13	-5.75	6.00
Roemer	1.515	4.170	-.12	1.66	-3.99	4.88	-.05	.07	.19	.11	.05	.08	10.08*	4.87
Samsom	-1.495	0.900	-2.09	3.44	.35	5.49	-.01	.12	.04	.17	.07	.14	3.75	5.86
Wilders	-1.279	1.295	-1.57	2.27	-1.00	14.00	-.08	.07	-.06	.13	.07	.08	24.37	16.20
Pechtold	1.907	4.599	.11	2.23	7.85	4.95	-.09	.11	.21	.14	.13	.13	-5.95	4.87
Haersma Buma	1.304	3.996	-.73	2.99	4.18	8.18	-.21	.12	-.05	.14	.10	.13	2.58	11.44

Notes: Δ AIC / Δ BIC: difference with the unstructured model (positive number is better fit).

*p < .05.

### 4. Perceiver sex effects

We expected that men would make larger formidability estimations than women would make, and we asked for participants’ self-reported height and weight to test whether these sex differences could be due to individuals using their own height and weight as anchors.

#### Height

Our tenth prediction (P10) stated that men would generally make larger estimations of height than women would make. Three analyses of covariance (ANCOVAs) showed that participant sex only had a robust significant effect on estimated height for the silhouettes measurement (3), and not on the cm measurement (1) or the slider measurement (2): (1) *t*(1959) = -1.649, *p* = .099, η_p_^2^ = .001, 95% CI [-.90, .08]; bootstrap: *p* = .095, 95% CI [-.90, .03]; (2) *t*(1969) = -.494, *p* = .621; (3) *t*(1969) = -2.38, *p* = .017, η_p_^2^ = .003, 95% CI [-.27, .03]; bootstrap: *p* = .010, 95% CI [-.27, -.02]. When estimating height with the silhouettes, men (*M* = -.06, *SD* = 1.40) estimated the party leaders to be shorter than women (*M* = .06, *SD* = 1.36) did.

#### Strength

Our eleventh and final prediction (P11) stated that men would generally make larger estimations of strength than women would make. Two analyses of covariance (ANCOVAs) showed that participant sex did not have a significant effect on estimated strength via sliders, *t*(2011) = -1.524, *p* = .128; or via silhouettes, *t*(2011) = -1.402, *p* = .161.

### Exploratory analyses

#### Participant sex effects on estimated weight

Two analyses of covariance (ANCOVAs) showed that participant sex did not have a significant effect on estimated weight in kg, *t*(1953) = -1.234, *p* = .218; or via sliders, *t*(1958) = .870, *p* = .384.

#### Participant height and weight effects on estimated height and weight

Three analyses of covariance (ANCOVAs) showed that participants’ own height only had a significant effect on estimated height for the slider measurements: *t*(1969) = -2.183, *p* = .029, η_p_^2^ = .002, 95% CI [-1.31, -.07]; bootstrap: *p* = .030, 95% CI [-1.30, -.04], and not in the cm measurements, *t*(1959) = 1.023, *p* = .306; or the silhouette measurements, *t*(1969) = -1.036, *p* = .300. When estimating height with the sliders, shorter participants estimated the party leaders to be taller than taller participants did.

Two analyses of covariance (ANCOVAs) showed that participant weight had a significantly positive effect on estimated weight, but only for the kilogram measurements: *t*(1953) = 3.543, *p* < .001, η_p_^2^ = .006, 95% CI [.25, .86]; bootstrap: *p* = .002, 95% CI [.19, .90], and not the slider measurements, *t*(1958) = -1481, *p* = .630. When estimating weight in kilograms, heavier individuals estimated the party leaders to be heavier than less heavy individuals did.

We have summarized our results in Table 17.

**Table 17:** Summary of results.

Prediction	Measurement	Supported?
P1		3/3
	Height cm	Yes
	Height sliders	Yes
	Height silhouettes	Yes
P2		2/2
	Strength sliders	Yes
	Strength silhouettes	Yes
P3		2/2
	Weight kg	Yes
	Weight sliders	Yes
P4		3/3
	Height cm	Yes
	Height sliders	Yes
	Height silhouettes	Yes
P5		1/2
	Strength sliders	Yes
	Strength silhouettes	No
P6		2/2
	Weight kg	Yes
	Weight sliders	Yes
P7		2/3^a^
	Height cm	Partially^a^
	Height sliders	No
	Height silhouettes	Partially^a^
P8		1/2^a^
	Strength sliders	No
	Strength silhouettes	Partially^a^
P9		2/2^b^
	Weight kg	Yes^b^
	Weight sliders	Yes
P10		1/3
	Height cm	No
	Height sliders	No
	Height silhouettes	Yes
P11		0/2
	Strength sliders	No
	Strength silhouettes	No

Note:

^a^ Only a loser-effect for Samsom.

^b^ We did find a ‘loser-effect’ of weight in kg for Samsom.

## Discussion

We hypothesized that distortions in judged formidability related to social status are the result of motivated social perception in order to promote group functioning and leadership. We expected that in-group leaders would be estimated as more formidable than out-group leaders, that prestige (measured through estimated performance) would have a positive effect on these formidability measurements, that winners would be overestimated and losers underestimated, and that men would make larger formidability estimations than women would. Our results partly supported and partly contradicted our expectations.

We expected in-group leaders to be overestimated more than out-group leaders would be. Our results showed significant positive effects of voting intentions on estimated height and strength, and these effects were fully or partially mediated by estimated political skills. This suggests that in-group leaders were not so much estimated as more formidable, but estimated as having better political skills, which in turn led to higher estimations of physical formidability. This also suggests that perceptual distortions of physical formidability are not just a heuristic, but rather the result of a motivated social perception: the association between status and size was stronger when it was beneficial for the individual, namely when estimating in-group leaders. These findings are in line with earlier research showing that presidential candidates were estimated to be taller by their supporters, as opposed to their opponents [55].

Our most robust finding deals with the expected positive effect of prestige on formidability estimations: estimated performance had a positive effect on all three estimated height measurements. Prestige also had a positive effect on the estimated strength measurement in sliders, but not for the silhouettes. As expected, prestige did not have a positive effect on the estimated weight measurements (as these do not contribute to a party leader’s physical formidability). We controlled for the possible effects of liking and age on the physical formidability measurements, and indeed found some effects: liking was positively associated with the height measurements in centimeters and via sliders and both strength measurements, and age was positively associated with both weight measurements. To our knowledge, this is the first study demonstrating that the status-size association is also driven by prestige, and suggesting that individuals have a motivated perception to see competent leaders as more formidable.

We expected men to make larger estimations of physical formidability than women would. However, if anything, results indicated the opposite effect. Participant sex only had a significant effect on height estimations via silhouettes, indicating that men typically made *smaller* estimations than women did. There was no significant effect of participant sex on any of the other four estimations of physical formidability, nor on the two weight estimations. This is in contrast to earlier findings suggesting that men made larger estimations of height (in feet and inches) of presidential candidates than women did [63]. Higham and Carment [63] explained this finding by suggesting that perceivers use their own height as an anchor for making estimations about the height of others (their research did not include actual height measurements or self-reported height of their participants). If this was indeed the case, then using continuous measurements (like our sliders and silhouettes) should reduce this effect. Moreover, we should also have found a positive effect of participant height (and weight) on the height (and weight) measurements, specifically the estimations in centimeters (and kilograms). Again, however, our findings contradict this argument, as participant height was actually, generally, negatively associated with estimated height (although we found one positive association between participant weight and estimated weight). Thus, the argument that men use their own (greater) height as anchor is not supported by these results. As men did not make larger estimations of physical formidability than women did, the competing sensitivity to opponents’ physical formidability theory is also not supported.

Finally, we tested winner/loser effects using a within-subject design. We expected that winners would be estimated as more formidable after the elections compared to before the elections, and losers to be estimated as less formidable after the elections compared to before the elections. In contrast to earlier work [55, 63] we did not find substantial evidence for a ‘winner effect’ in our study. Samsom was estimated less tall (in two out of three height measurements) after the elections compared to before. In contrast to our hypothesized non-effects on the weight measurements, Samsom was also estimated less heavy (in the kg measurement, but not the slider measurement) after the elections compared to before. Against our expectations, he was also estimated as stronger after elections compared to before the elections (in the strength via silhouettes measurement, but not the slider measurement). The significant time effects we found for van Haersma Buma were generally in the expected direction: van Haersma Buma, who lost eight seats, was estimated shorter (via silhouettes) and less strong (via sliders) after the elections compared to before the elections. However, there were also some effects that were not in line with our expectations. Wilders, who lost nine seats, was estimated as taller (via silhouettes) after the elections compared to before the elections. Surprisingly, Roemer, Samsom, and van Haersma Buma were all estimated stronger after the elections compared to before the elections in the strength via silhouettes measurements. This could be due to the used stimulus images, as none of the depicted bodies accurately matched the average politician’s body type (Fig 2). This notion is strengthened by the fact that for Samsom and van Haersma Buma, this was the only measurement that generated opposite results compared to the other formidability measurements. Moreover, effect sizes of the strength slider measurement were particularly large compared to the other height and strength measurements.

Overall, we can conclude that Samsom was estimated as less formidable after losing the prime-ministership, indicating a loser effect. This is in line with earlier research showing that presidential candidates who lost an election were estimated shorter compared to before the elections [55,63]. We should note that, possibly due to the used stimulus images, results of one of the strength measurements suggested an opposite effect. A methodological strength of our study is that we used multiple measures for height, strength, and weight, which allows us to both test the robustness of found effects, as well as make comparisons between different types of measurements. Furthermore, we used a more critical within-subject design, comparing estimations of participants individually, as opposed to a between-subject design, which only compares group means. Another strength is that we studied a multi-party European country, adding knowledge to the research pool that until now mostly consisted of North-American studies. However, the Dutch political system does complicate the definition of winners versus losers compared to a simpler two-party presidential election. For example, even though a politician might have gained a substantial number of seats, he/she might have lost the highest office (prime-ministership). In our study, the majority of the participants indicated that Samsom was the greatest winner after the elections (however very closely followed by Rutte). Interestingly, the majority of the participants also voted for Samsom: while explicitly still supporting their leader, implicitly they did estimate Samsom as less formidable after the elections. Further research could focus more on the possible difference between explicit and implicit perception measures after winning or losing a competition.

There are some limitations to our study. Although we found overall effects of prestige estimations on formidability estimations, occasionally individual politicians stood out. These findings can possibly be explained by the deviations in height and weight that these individuals have (see also [55]). These more extreme builds likely leave less room for perceptual distortion. Furthermore, we used a self-selected sample in this study: visitors of a website with voting advice were invited to take part in the study. This method has two advantages: 1) it has given us the opportunity to test our hypothesis within a large number or participants; and 2) it has given us the opportunity to examine real-life coalitional affiliations, as participants were actually planning to vote on these leaders or their opponents during election times. However, a disadvantage of this method is that it could lead to a biased sample, as certain participants were likely more willing to complete the survey than others. Indeed, in our sample, left-wing party voters were somewhat overrepresented, while right-wing party voters were somewhat underrepresented (Table 1). Moreover, visitors of the website are likely to be more interested in politics than the typical citizen.

Interestingly, different types of measurement (for example asking estimations of height in centimeters, sliders, and via silhouettes) sometimes lead to different results. Overall, it seems like continuous measurements in sliders give more ‘opportunity’ for perceptual distortions than more concrete measurements like centimeters or kilograms do. Moreover, although the height silhouettes seemed to be a representational stimulus, the strength silhouettes were possibly not a good choice in the context of our third study. For future research, it should be noted that the stimulus used to measure formidability estimations could have an impact on the outcomes, as at least in one case we found opposing effects depending on the choice of silhouette vs. slider measure.

Our results suggest that individuals associate prestige with formidability, and even though these motivated perceptions can be beneficial for leadership or group functioning, they also have a downside. One implication of the findings is that these kinds of cognitive biases could have negative effects for short men, as they could be inaccurately seen as less competent, even when there is no evidence that size matters for performance, while tall men could be undeservedly seen as more competent. Along these lines, Judge and Cable [11] even suggested that the social esteem that comes with being tall might lead to a ‘halo-effect’ of height with constant positive feedback of others leading to actual higher self-esteem and self-worth in taller individuals [76]. This leads to a self-fulfilling prophecy. Shortness, on the other hand, could actually become a liability, as it is not associated with high status positions [16].

We conclude that the positive association between status and size is more than just a mere heuristic, but the result of a motivated perception of leaders that could be beneficial for group functioning. Moreover, size is linked not only to status in terms of dominance, but also in terms of prestige. Our data do not support any perceiver sex or winner effects of perceived formidability. However, they do suggest a negative loser effect. More research on this topic is needed to further explore these associations and researchers should keep in mind that different means of perceived formidability measurements could lead to different outcomes.

## Supporting information

S1 File Data part one. (SAV)

S2 File Data parts one and two merged. (SAV)

S3 File Electronic Supplementary Materials. (DOCX)
