# Supplementary material for: Size, skills, and suffrage: Motivated distortions in perceived formidability of political leaders
Source: PLoS One. 2017 Dec 21;12(12):e0188485. doi: 10.1371/journal.pone.0188485 (PMC5739394; doi:10.1371/journal.pone.0188485)
Supplement: S3 File — (DOCX) [file pone.0188485.s003.docx]

**ESM Size, Skills, and Suffrage: Motivated Distortions in Perceived Formidability of Political Leaders**

**Accuracy of height estimations**

On average, participants were not able to correctly estimate the party leaders’ heights in centimeters, however some leaders were better estimated than others ((Table 2; six one-sample t-tests showed that all average height estimations significantly differed from the actual heights (all *p’*s < .001)). In line with previous research [63], most party leaders’ heights were underestimated, except for Samsom and van Haersma Buma, who were overestimated. Samsom and van Haersma Buma were also the only two leaders who were reasonably accurately estimated, as their estimated heights differed < 3 cm from their actual heights.

**Effects of control variables**

**Height – cm measurements**

Liking also had a significant effect on estimated height: when party leaders were better liked, they were also estimated to be taller (*t*(1959) = 2.836, *p* = .005, η_p_^2^ = .004, 95% CI [.01, .04]; bootstrap: *p* = .019, 95% CI [.00, .­04]). However, estimated age did not have a significant effect on estimated height (*p* = .978).

**Height – slider measurements**

Liking and estimated age also had a significant effect on estimated height. When party leaders were better liked, they were also estimated to be taller (*t*(1969) = 4.807, *p* < .001, η_p_^2^ = .012, 95% CI [.05, .13]; bootstrap: *p* = .001, 95% CI [.05, .­13]). When party leaders were estimated to be older, they were also estimated to be taller (*t*(1969) = 2.054, *p* = .040, η_p_^2^ = .002, 95% CI [.00, .12]); however the bootstrapped confidence interval suggests this is not a robust effect: *p* = .051, 95% CI [-.00, .­12]).

**Height – silhouette measurements**

Liking also had a significant effect on estimated height: when party leaders were better liked, they were also estimated to be taller (*t*(1969) = 4.355, *p* < .001, η_p_^2^ = .010, 95% CI [.00, .01]; bootstrap: *p* = .001, 95% CI [.00, .01]). estimated age (*p* = .397) did not have a significant effect on estimated height via silhouettes.

**Strength – slider measurements**

Liking also had a significant effect on estimated strength: when party leaders were better liked, they were also estimated to be stronger (*t*(2011) = 14.070, *p* < .001, η_p_^2^ = .090, 95% CI [.24, .31]; bootstrap: *p* = .001, 95% CI [.24, .32]). Estimated age (*p* = .195) did not have a significant effect on estimated strength.

**Strength – slider measurements**

Only liking (*t*(2011) = 5.053, *p* < .001, η_p_^2^ = .013, 95% CI [.01, .01]; bootstrap: *p* = .001, 95% CI [.00, .01]) had a significant effect on estimated strength. When party leaders were better liked, they were also estimated to be stronger. estimated age (*p* = .463) also did not have a significant effect on estimated strength.

**Weight – kg measurements**

Furthermore, estimated age also had a significant effect on estimated weight: when party leaders were estimated to be older, they were also estimated to be heavier (*t*(1953) = 3.416, *p* = .001, η_p_^2^ = .006, 95% CI [.02, .08]; bootstrap: *p* = .001, 95% CI [.02, .08]). Liking did not have a significant effect on estimated weight (*p* = .474).
**Weight – slider measurements**

Only estimated age had a significant effect on estimated weight: when party leaders were estimated to be older, they were also estimated to be heavier (*t*(1958) = 3.251, *p* = .001, η_p_^2^ = .005, 95% CI [.04, .17]; bootstrap: *p* = .003, 95% CI [.04, .16]). Liking had a marginally significant effect on estimated weight (*t*(1958) = 1.924, *p* = .055, η_p_^2^ = .002, 95% CI [-.00, .08]; bootstrap: *p* = .073, 95% CI [-.00, .08]); however the confidence interval suggests this is not a robust effect.
